# Exploring the role of AI algorithmic agents: The impact of algorithmic decision autonomy on consumer purchase decisions

**DOI:** 10.3389/fpsyg.2022.1009173

**Published:** 2022-10-20

**Authors:** Yuejiao Fan, Xianggang Liu

**Affiliations:** Business School, Huaqiao University, Quanzhou, China

**Keywords:** AI algorithmic marketing, algorithm agent role, algorithmic decision autonomy, self-efficacy, power distance, purchase decisions

## Abstract

Although related studies have examined the impact of different images of artificial intelligence products on consumer evaluation, exploring the impact on consumer purchase decisions from the perspective of algorithmic decision autonomy remains under-explored. Based on the self-determination theory, this research discusses the influence of the agent decision-making role played by different AI algorithmic decision autonomy on consumer purchase decisions. The results of the 3 studies indicate that algorithmic decision autonomy has an inverted U-shaped effect on consumer’s purchase decisions, consumer’s self-efficacy mediates the relationship between algorithmic decision autonomy and purchase decisions, and consumer’s power distance moderates the relationship between algorithmic decision autonomy, self-efficacy, and purchase decisions. The research results can provide references for marketers, retailers, algorithm designers, and other parties to formulate algorithm marketing strategies, make AI algorithm decisions better serve consumers, and achieve value co-creation with consumers.

## Introduction

In the era of the digital economy, data have become a strategic factor of production involved in the whole process of value creation, distribution, circulation, and consumption, while algorithms have become a strategic tool for collecting and processing data, resulting in AI algorithms decision-making based on data elements (Logg et al., 2019; Hoffman et al., 2022). Compared with human decision-making, algorithmic decision-making has the advantages of being fast, pervasive, and low consumption (Bonnefon et al., 2016). With these characteristics, algorithms have been becoming the basis of decision-making in “algorithmic life” and have started to intervene and even dominate more and more human social affairs, becoming agents in people’s daily lives (Bo and Benbasat, 2007; Danaher et al., 2017). For example, many of the content services that people access on the Internet, such as news, music, video, advertising, social network dynamics, and the goods they buy, are currently personalized by recommendation engines based on consumer preferences, not by human decisions (sohu.com)^1^ (Gal, 2018). Most Internet technology companies are already using algorithmic decision-making in consumer, education, finance, healthcare, transportation, justice, urban governance, and other fields and scenarios (Mestel et al., 2018; Dubey et al., 2021). For companies using algorithms for decision-making, algorithms are not only marketing or sales tools, but also an important driving force to stimulate insight, innovation, and user participation. Therefore, with the popularity of algorithms in consumer-oriented decision-making, it is of great significance to understand how consumers react to algorithmic decisions (Hoffman et al., 2022; Yalcin et al., 2022).

A review of the relevant literature reveals that most of the existing studies on algorithms have focused on technical improvements in human-computer interaction or investigated the influence of algorithm-related features on people’s motivation to accept (Logg et al., 2019), or compared algorithmic decisions with human decisions to derive the reasons and factors influencing preferences (Dietvorst et al., 2015; Dietvorst and Bharti, 2020; Yalcin et al., 2022), while studies exploring the interactive processes and psychological mechanisms of consumer-algorithmic decision-making are relatively limited, and the impact of algorithmic decision autonomy on consumer purchase decisions and its mechanisms of action from the perspective of algorithmic decision autonomy has not been studied yet (Paschen et al., 2019). Existing research reviews on the impact of algorithmic decision-making are mixed and have yet to reach a consistent conclusion. On the one hand, scholars argue that sophisticated algorithms allow online marketers to offer just the right product or service that not only alleviates consumers’ search costs but also alleviates the trade-off of difficult choices and increases the utility they derive from their choices. On the other hand, scholars have argued that algorithmic decision-making can undermine consumers’ sense of autonomy and may be harmful to consumers’ well-being (André et al., 2018), and even cause algorithmic pollution (Marjanovic et al., 2021). This kind of decision-making based solely on interest-based algorithmic recommendations limits users’ access to diverse information and ties them to the “echo chamber” built by AI algorithms. So, while algorithms can often make more accurate decisions than humans, people still prefer human decisions. Therefore, we aim to address these research questions:

1. What role do AI algorithmic agents play in social interactions with consumers?
2. Is there an optimum for the influence of algorithmic decision autonomy on consumer decisions?
3. What are the cognitive processes and conditions for the impact of algorithmic decision autonomy on consumer decisions?

**Figure 1:**
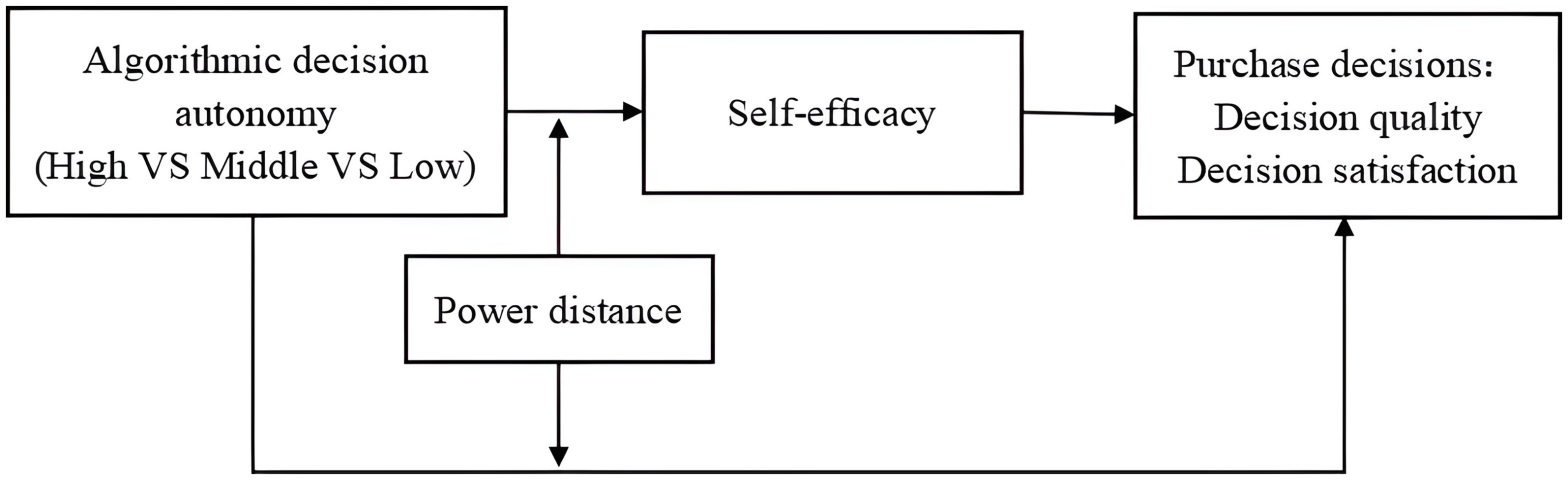
Theoretical framework.

To answer these research questions, based on self-determination theory, we construct a model framework (as in Figure 1) to describe the causal mechanism between algorithmic decision autonomy and consumer purchase decisions, and classify algorithmic decision autonomy into three levels (high vs. middle vs. low) based on the relationship between algorithms, human, and society, which correspond to the corresponding agent roles: dictatorial substitute/co-assistant/pure performer. Three studies are conducted to explore the effects of algorithmic decision autonomy on consumer purchase decisions; the mediating effect of consumer self-efficacy in algorithmic decision autonomy and purchase decisions; and the moderating role of the heterogeneous characteristics of consumer power distance (high vs. low).

## Theoretical background and hypothesis development

### Self-determination theory

Self-determination theory is a motivational theory of human behavior that investigates the extent to which individuals are self-determined, as reflected in the three major psychological needs motivating consumer autonomy, competence, and relatedness (Brown and Ryan, 2003). Autonomy represents the ability of an individual to make choices and determine a course of action based on his or her own volition, without external coercion (e.g., initiating, regulating, and maintaining his or her behavior; Lau and Ki, 2021). The need for competence reflects the desire to interact effectively with the environment, and when this need is met, people have a sense of control and accomplishment (Puntoni et al., 2021); Relatedness needs refer to people’s need for mutual respect and connection with others, and when this need is met, people will feel social support from others and enhance their sense of social existence (Deci and Ryan, 2000).

According to SDT, the degree of algorithmic decision autonomy affects these three major intrinsic motivations of consumers. Firstly, when the degree of algorithmic decision autonomy is high, it can deprive consumers of autonomy (Puntoni et al., 2021), because consumers feel that they experience systematic discrimination or oppression, are socially excluded, and are limited in the expression of their own autonomous needs (Kachanoff et al., 2020), thus influencing their purchasing decisions. Secondly, users do not simply receive algorithmic output but will process the concept of algorithmic information they receive, and they will rely more on their intuition than algorithmic decisions in the decision-making judgment process (Shin, 2022), this is a demonstration of their competence. Thirdly, in terms of relatedness, humans perceive themselves as distinct from other groups, but the control of the environment by algorithmic decisions, especially those with high autonomy, may blur the boundary between “human” and “tool” and bring about the annihilation of human uniqueness, threaten human identity or uniqueness and lose their sense of control (Faraji-Rad et al., 2017; Longoni et al., 2019), which is detrimental to consumers’ purchasing decisions (Kim et al., 2015; Lau and Ki, 2021). In addition, it has also been shown that self-determination allows people to feel that they have control over their choices and lives, which ultimately increases their feelings of psychological wellbeing (e.g., feeling capable, self-governed, well-supported, and satisfied with their state; Brown and Ryan, 2003) Therefore, SDT explains well the influence mechanism of algorithmic decision autonomy on consumer purchase decisions (Beer, 2017).

### Algorithmic decision autonomy

The degree of algorithmic decision autonomy reflects the extent to which AI decision systems based on big data and machine learning and deep learning algorithms operate in an independent and goal-directed way without users’ interference (Baber, 1996; Rijsdijk et al., 2007; Rijsdijk and Hultink, 2009), takes over tasks on its own and can exhibit proactive and self-initiated behavior (Benlian et al., 2020; Lucia-Palacios and Pérez-López, 2021). While human autonomy refers to individuals’ ability to carry out a decision that fits their needs or desires free from coercion or manipulation (Dogruel et al., 2022), which is associated with self-determination theory. Humans have been rational and ethical decision-makers for thousands of years, but emerging algorithmic technologies are now replacing human autonomy by making decisions for humans (Chiodo, 2022), which can threaten the autonomy of human decision-making (Burton et al., 2020; Dogruel et al., 2022). At the same time, algorithmic decision-making may challenge the control of human decisions, where potential biases, discrimination, censorship, or the emergence of echo chambers may occur (Dogruel et al., 2022; Shin et al., 2022c). Autonomous algorithms may also collect information from the environment about the consumer’s behavior, and share this information with third-party service providers without the consumer’s permission (Shin et al., 2022d), raising a range of issues such as transparency, fairness, accountability, and explainability (Shin, 2021; Shin et al., 2022c). Therefore, AI algorithm decision-making involves the decision-making mechanism of human and machine interaction, which is reflected in the relationship between human, algorithm and society. The most basic and core is the autonomy relationship between human and algorithm in decision-making, which is reflected in the proportional relationship between them, or in other words, the relationship between the two is a trade-off (Hongjun, 2022).

There is no unified knowledge about algorithmic decision autonomy and the role it represents, but we can draw insights from a series of studies by many scholars or institutional subjects. According to the order of development and degree of intelligence of AI, Huang et al. distinguished four types of intelligence: mechanical, analytical, intuitive, and empathic (Huang and Rust, 2018). After that he merged both analytic and intuitive AI, forming mainly three types of AI, which are mechanical, thinking, and feeling AI (Huang and Rust, 2021a), and was widely cited (Huang and Rust, 2021b; Pantano and Scarpi, 2022; Schepers et al., 2022). Some studies classify AI into three categories: narrow AI, general AI, and super AI (Kaplan and Haenlein, 2019; Benbya et al., 2020; Ameen et al., 2022). Based on the different roles of algorithmic decision agents, OECD (2017) also proposed four types of roles for AI algorithms: Monitoring algorithms, Parallel algorithms, Signaling algorithms, and Self-learning algorithms. But parallel algorithms and signaling algorithms are decisions that are ultimately made by humans and can be grouped into the same category. Therefore, from the perspective of the human-algorithm decision relationship, the above division of AI types actually corresponds to three different degrees of autonomy of AI algorithms: high, middle, and low (Leung et al., 2018; Dogruel et al., 2022), and play different roles in the social value network (Čaić et al., 2018). When the algorithm has absolute dominance in decision-making, the “autonomy” of the algorithm will be at the highest level and the algorithm becomes an independent decision-maker; when the human has dominant autonomy in decision-making, the “autonomy” will be close to the middle level and the algorithm is an auxiliary decision-maker in the human-algorithm relationship; and when the “autonomy” of the algorithm is at a lower level, the algorithm becomes a mechanical executor in the human-algorithm relationship (Leung et al., 2018). According to SDT, these three different levels of autonomy and the corresponding roles of decision-making agents have different effects on consumer’s psychological and behavioral activities. Research shows that, at low levels of autonomy, one AI product is unable to act by itself and start listening without the user’s interaction, and that, as autonomy increases, the benefits can outweigh the risks until to the point that increasing autonomy leads to a loss of control (Lucia-Palacios and Pérez-López, 2021). Huang et al. also argue that substitution effects in decision-making are more likely to occur at high levels of AI (Huang and Rust, 2018).

### Algorithmic decision autonomy and purchase decisions

Consumer purchase decisions are influenced by multiple factors, and the decision process is often intertwined with risk perception, emotions, cognition, etc. Therefore, from a customer journey perspective, decision quality and decision satisfaction are two important measures of decision outcomes (Bo and Benbasat, 2007). Bechara et al. (1998)believe that decision-making is not only a cognitive process but also a process of cognitive processing after inputting a large amount of information such as feedback related to emotion or motivation. Kuo et al. (2009)have shown that information representation can cause emotional changes, which can change cognitive strategies, which in turn can affect decision-making behavior. Therefore, our studies focus on the impact of individual consumer level factors and psychological cognitive processes on their decision quality and satisfaction in the context of AI marketing.

When the algorithm becomes an independent decision-maker in the relationship between people and algorithms, that is, the algorithm has a high degree of decision-making autonomy and human autonomy is relatively small. According to SDT, algorithms intervene too much in the human operational process, which creates the negative potential hazard of algorithmic overdependence, which will reduce human satisfaction with the operational process and deepen systematic bias against AI algorithms (Banker and Khetani, 2019). Because individuals feel that their behavior is controlled by AI algorithms, their autonomous needs cannot be satisfied, which leads to a decrease in internal motivation, and the independent decision-making of the algorithm makes consumers perceive that their freedom of decision-making and autonomy is violated, which leads to resistance (André et al., 2018). The most typical example is that information delivery platforms are using recommendation algorithm technology to subvert people’s reading in this era, mastering the voice power of news information distribution, people’s reading selection decisions are determined by algorithms. Even worse, companies are using AI algorithms to manipulate public opinion. In addition, according to the “uncanny valley” theory (Stein and Ohler, 2017), the autonomous decisions by algorithms can be disconcerting, and people’s reactions can change from empathy to aversive and fear, which may inhibit consumers’ perceptions of how willingness to adopt algorithms affects their decision quality and decision satisfaction (van Doorn et al., 2017), creating algorithmic decision loss aversion (Kim et al., 2019). Therefore, the following hypotheses are framed:

*H1a:* When algorithmic decision autonomy is high, it will have a negative impact on consumers’ purchase decisions.

When humans have relatively dominant autonomy in decision-making, the autonomy of algorithmic decision-making is close to the middle level, and the algorithm plays an auxiliary role in the human-algorithm relationship. In this context, algorithmic decision-making can well improve the efficiency of decision-making, provide an important reference for consumer decision-making with more accurate services (Prahalad and Ramaswamy, 2004; Lalicic and Weismayer, 2021; Yin and Qiu, 2021), and will not undermine consumers’ decision autonomy. According to self-determination theory, when the content of algorithmic decisions based on users’ needs and preferences satisfies their unique preferences. Users not only feel pleasure and satisfaction, and their loyalty will be improved to a certain extent, but also enhance their perception of reaching consensus on their decision-making and recommender system, generating stronger confidence in their choices, and thus satisfaction with the algorithmic decisions (Xiao and Benbasat, 2018). Meanwhile, personalized recommendations make it easier for users to access information of interest and better products and services, choices become easier, practical, and effective, and decision quality is improved, so users develop positively perceived usefulness and perceived personalization (André et al., 2018; Palos-Sanchez et al., 2019), increasing their cognitive and affective trust, and thus a significantly higher willingness to adopt algorithmic decisions (Palmeira and Spassova, 2015; Starke and Lünich, 2020), which contributes to enhance individuals’ internal motivation for the decision task and improve the sense of social presence. Accordingly, we hypothesize the following:

*H1b:* When the algorithm decision-making autonomy is at the intermediate level, this is the most comfortable agency relationship, which has the highest impact on consumers’ purchase decisions.

When the algorithm has no autonomy in decision-making, and the autonomy of the algorithm is at the lowest level, the algorithm becomes a “pure executor” in the human-algorithm relationship. Although the degree of self-determination reaches the highest, everything needs to be decided by consumers, just a pure executor will affect the user experience, affect the efficiency of decision-making, and do not provide the necessary decision-making reference, which is tantamount to increasing the difficulty of decision-making and will cause consumers to decision boredom, which is contrary to the trend of digital advancement (Yunwen, 2021). In addition, when the algorithm becomes a pure executor, there may be great problems in the practice of algorithm decision-making, because the past data may be outdated, and people’s preference characteristics may change over time. However, the algorithm decision-making that blindly implements “poor historical data” will strengthen the past shortcomings and accumulate the adverse effects of bias disadvantages (Marjanovic et al., 2021), reflecting the non-intelligence of algorithmic decisions that do not change iteratively with the environment. Based on SDT, when AI algorithms have low decision autonomy, they are characterized as mechanical, rigid, and inflexible, and will only do standardized and repetitive tasks according to a set procedure (Longoni et al., 2019). Algorithmic decision-making suffers from a lack of flexibility and does not satisfy consumers’ motivation for unique needs. Consumers will tend to find it difficult to interact with AI algorithmic systems or products. Based on the points discussed above, we propose the following hypothesis:

*H1c:* When the algorithmic decision autonomy is at a low level it has a negative impact on the consumer’s purchase decision.

*H1:* Combining these three levels, algorithmic decision autonomy has an inverted U-shaped effect on consumers’ purchase decisions.

### Mediating role of self-efficacy

Self-efficacy is defined as the degree of one’s feelings about his/her ability to accomplish goals (Bandura, 1977). In the context of algorithms, extensive research has shown that user’s self-efficacy is significantly associated with algorithmic decisions. Shin et al. studied the positive effect of perceived fairness, explainability, accountability, and transparency on users’ self-efficacy of personalized algorithms (Shin et al., 2022a,b). Hu et al. confirmed that the sensing, thought and action autonomy of artificial intelligence has a positive impact on the competence and warmth perception of individuals (Hu et al., 2021). It has also been shown that better learning of algorithmic skills in a learning environment can improve algorithmic thinking and achieve higher levels of self-efficacy (Fanchamps et al., 2021). Based on SDT, individuals generally want to achieve control over the external environment, while algorithmic decision-making is conducive to the realization of this goal to a certain extent. Because in an environment where the purchase task makes a decision, it is actually a depletion of the consumer’s cognitive resources, while the algorithm as a decision tool contributes to the consumer’s motivation to mobilize, cognitive resources, and ability to take actions to successfully complete the task in the decision process (Orth and Wirtz, 2014; Wang and Jiang, 2022). Given this, H2 is proposed:

*H2:* Algorithmic decision autonomy has a positive effect on consumer self-efficacy.

Self-efficacy plays a key role in how humans perform because it directly influences factors such as motivations and goals, affective tendencies, perceptions of opportunities, and outcome expectations in social environments. Research suggests that consumer self-efficacy may affect their decision-making, the greater the consumer’s self-efficacy for decision-making tasks, the more efficient the decision-making process strategies are expected to be (Hale et al., 2021). When self-efficacy is considered in terms of people’s online behavior, the higher the online self-efficacy, the more confident people are in using the algorithm (Araujo et al., 2020). Further, the greater the individual’s belief in their online self-efficacy, the higher the individual’s positive attitude toward the use of algorithmic decision-making (Mahmud et al., 2022). Self-efficacy can increase the adoption of both sustainable behaviors, such as fintech usage intentions (Lee, 2021), recycling intentions (White et al., 2019), and health behaviors (Han et al., 2016). Lastly, self-efficacy can benefit one’s psychological well-being. For example, feelings of self-efficacy associated with self-made creations were found to produce positive feelings as well as a greater willingness to pay for the product (Cannon and Rucker, 2022). Consequently, the following hypotheses are framed:

*H3:* Self-efficacy has a positive effect on consumer purchase decisions.

*H4:* Self-efficacy mediates the inverted-U relationship between algorithmic decision autonomy and purchase decisions.

### Moderating of power distance

Power distance reflects the individual’s cultural values about society, and refers to people’s acceptance and expectation of the uneven distribution of power (Hofstede, 1980; Oyserman, 2006). Research has shown that consumers with different power distance perceptions have different preferences for decision-making, which can influence people’s attitudes and behaviors. Consumers with low power distance perceive equality everywhere in their lives, and they are committed to respecting the equality that exists in social communication processes (Gao et al., 2016), they tend to cooperate with others. Algorithmic decision-making brings them convenience in decision-making, do not have algorithmic decision aversion even when there is a high level of algorithmic decision autonomy. Because they are affectionate and forgiving (Han et al., 2017), they are more likely to accept even if the algorithm is biased. In contrast, people with high power distance have a greater sense of status (Kim and Zhang, 2014) and self-confidence, and pay attention to their emotional self-evaluation of being socially accepted (Soll and Mannes, 2011). Therefore, when the degree of algorithm decision-making autonomy is high, it will make them lose the evaluation degree of self-determination and reduce their sense of self-efficacy.

At the same time, people with high power distance will have reduced motivation to connect with others while maintaining social distance with others. According to the self-determination theory, high power distance people prefer to self-judge and self-determine and self-make decisions than low power people and thus feel bad about algorithmic decision-making especially when algorithms become independent players. Typically, as executives with a high sense of power distance, they have long resisted the use of AI decision-making for higher-level decision-making. They always prefer intuitive decision-making based on field experience rather than AI-assisted decision-making (Banker and Khetani, 2019; Thurai and McKendrick, 2022). Research shows that people who strongly identify with a specific social category will resist the results of identity-related consumption algorithm autonomous decision-making, because they have a higher degree of attribution identification for their internal motivation decision-making (Leung et al., 2018). Those with a high sense of power follow the dynamic orientation of power when making decisions for themselves, placing more value and importance on themselves and being more self-focused (Rucker et al., 2011). Thus, we hypothesize the following:

*H5:* Power distance plays a negative moderating role in the effect of algorithmic decision autonomy on purchase decisions.

*H6:* Power distance plays a negative moderating role in the effect of algorithmic decision autonomy on self-efficacy.

## Overview of studies

Three different study scenarios were manipulated to test the hypothesis. In study 1, taking the news information distribution platform as the background, a one-way between-group design (algorithm decision autonomy: high vs. middle vs. low) was used to test the inverted U effect of algorithm decision autonomy on consumers’ purchasing decisions, including H1a/b/c. In study 2, against the background of the decision to purchase a home AI service robot, a one-way between-group design was used to test H1 again and to test the mediating effect of self-efficacy, including H2/3/4. Study 3 used a 3 (algorithmic decision autonomy: high vs. middle vs. low) × 2 (power distance: high vs. low) between-group experimental design based on an AI shopping guide service program of an e-commerce platform, the aim is to test again the effect of the inverted U-shape and the moderating effect of power distance, i.e., H5/6.

### Study1: Main effect

#### Pre-study 1

##### Stimuli and design

The pre-experiment mainly tests the experimental situation, experimental method, and the validity of the scale. A total of 70 participants were recruited through the “credamo” online platform (The platform is equivalent to the Amazon “MTurk” portal and is used for online recruitment of willing research study participants). All subjects were randomly divided into three groups (News information distribution platform as the background (Shin et al., 2022d), algorithmic decision autonomy high vs. middle vs. low) of text material experimental scenarios. Subsequently, algorithmic decision autonomy was measured as manipulation tests, and a general judgment question on the role of the algorithmic agent “Based on the decision scenario, do you think its role belongs to a pure performer/co-assistant/dictatorial” (Lucia-Palacios and Pérez-López, 2021), and included attention questions is built to test whether subjects answer carefully. Finally, demographic information and familiarity were completed.

##### Results

The four items of autonomy had high reliability (*α* = 0.928), the number of subjects in the three groups was 23:24:23, and the results of the manipulation test showed that there was a significant difference in the subjects’ autonomy of algorithmic decision-making between the three groups (*F* (2, 67) = 9.133, *p* < 0.001). The cross-table analysis of the manipulation judgment questions of the experimental group and the algorithm agent role shows that participants with high autonomy in algorithmic decision-making preferred the role of “dictatorial substitute” (78.3%, *M* = 4.359, SD = 1.857); participants with moderate autonomy in algorithmic decision-making think the algorithm tends to play the role of “co-assistant” (87.5%, *M* = 4.271, SD = 0.992); participants with low autonomy think that the algorithm tends to be a “pure executor” (82.6%, *M* = 2.826, SD = 1.328), Pearson chi-square test and Monte Carlo two-tailed test were significant. *Post hoc* multiple comparison analysis Dunnett’s *t*-test (two-tailed) showed significant differences between the two groups (*p*_max_ = 0.002 < 0.05), indicating that the experimental manipulation test was successful and that the experimental context and information will be used in study 1.

#### Study 1

##### Design and procedures

In addition to adding two dimensions of consumer purchase decision quality and decision satisfaction measurement (Amason, 1996; Ameen et al., 2021), the procedures and contents of the formal experiment were the same as those of the pre-study. The measurement scales were all seven-point Likert scales. The formal experiment calculates the sample size in advance. Based on the calculation method and relevant research in Cohen (1977), G*power is used to set the statistical power in advance (*A priori*) to determine the sample size. The *F*-test, a one-way ANOVA statistical test was selected from the test set, and the effect size was set to a middle effect size of 0.25 (effect size *f* = 0.25), the *α* level of the two-tailed test was controlled at 0.05, and the expected efficacy value of 0.8 (power = 0.80), and then the number of groups 3 was entered to calculate the sample size should reach 159 or more. Therefore, the study recruited 160 consumers through the credamo, and participants were randomly assigned to 3 groups. The sample distribution of each group of the algorithmic decision autonomy was *N*_high_ = 53, *N*_middle_ = 54, *N*_low_ = 53; the proportion of women was 67.5%, the age group was concentrated between 18 and 50 years old (about 95%), and the education level was concentrated in undergraduate and high school and college level; in addition, most of the respondents (56.25%) were managers, students, clerks, and 77.5% had a monthly income of more than 3,000 RMB.

##### Results

ANOVA analysis results show that there are significant differences in the subjects’ decision-making autonomy of the three groups of algorithms (*F* (2, 157) = 49.946, *p* < 0.001; *M*_high_ = 5.160, *M*_middle_ = 4.579, *M*_low_ = 2.816), the homogeneity of variance was equal. The Dunnett’s *t*-test of multiple comparative analyses showed that there were significant differences between the two groups (*p* = 0 < 0.05). The results with purchase decisions as the dependent variable show that the effect of algorithmic decision autonomy on purchase decisions is significant (*F* (2, 157) = 19.210, *p* < 0.001), and the evaluative impact of consumer purchase decisions is the largest when the algorithmic decision autonomy is at a middle level, followed by the other two (*M*_middle_ = 5.625 > *M*_high_ = 4.580 > *M*_low_ = 4.250), verifying the H1b.

**Figure 2:**
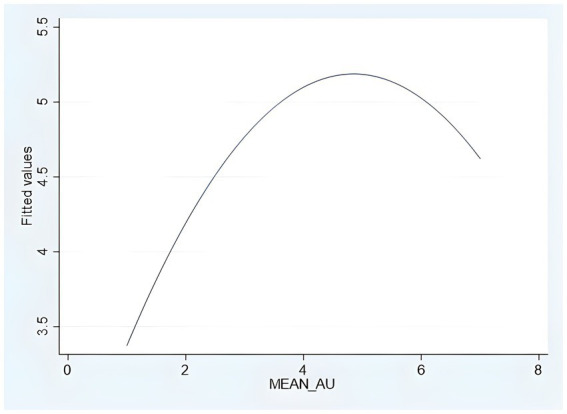
Inverted U-shaped curve fitting (Study 1).

Next, we verify the inverted U-shaped relationship presented by the influence of algorithmic decision autonomy on consumer purchase decisions. First, the regression estimation of the curve was fitted by SPSS 26, and the results showed that the quadratic and primary terms standardized regression coefficients of *β*_2_ = −1.213 (*t* = −2.757, *p* = 0.007) and *β*_1_ = 1.427 (*t* = 3.243, *p* = 0.001) were significant, while the cubic curve fitting was not significant, proving that the non-“S” curve and the inverted U-curve relationship regression equation can be obtained: *y* = −0.122 (*x*^2^) + 1.187*x* + 2.311. Second, the independent variable was squared and included in hierarchical regression, and Model 3 in Table 1 shows that the square of decision autonomy (*β*_2_ = −0.142, *p* < 0.01) is significantly and negatively related to the purchase decision. However, Haans et al. argue that significant coefficients alone are not sufficient to establish a quadratic relationship (Haans et al., 2016). Therefore, using their research methods on inverted U-shaped curves for reference, we use Stata to carry out the standardized “*U*” test (the fitted Figure 2 is as follows). The algorithmic decision autonomy ranges from −1.993 to 1.759, when at −1.993, the slope is 1.133, which is positive; at 1.759, the slope is −0.633, which is negative. After calculation, the turning point is 0.414, which is within the range of the algorithmic decision autonomy. Therefore, the hypotheses of H1a, H1b, H1c, and the inverted U-shape of H1 are fully verified.

**Table 1:** Regression results.

Variables	Purchase decisions (Study 1)			Purchase decisions (Study 2)				Self-efficacy (Study 2)		
	Model 1	Model 2	Model 3	Model 4	Model 5	Model 6	Model 7	Model 8	Model 9	Model 10
*Control variables*										
Gender	0.144	0.028	0.024	0.243	0.071	0.076	0.162	−0.03	−0.129	−0.129
Age	0.175	0.138	0.104	0.067	0.142	0.172	0.164^*^	−0.035	0.008	0.012
Education	−0.140	−0.102	−0.203	−0.284	−0.27	−0.242	−0.054	−0.295^*^	−0.286^*^	−0.283^*^
Occupation	−0.051	−0.043	−0.044	−0.022	−0.029	−0.033	−0.018	−0.017	−0.021	−0.022
Income	0.095	0.055	0.106	0.060	0.034	0.032	−0.013	0.083	0.068	0.068
Familiarity	0.003	0.009	0.086	0.123	0.088	0.121	−0.029	0.242^**^	0.221^**^	0.226^**^
*Independent variables*										
ADA		0.177^**^	1.327^**^		0.398^***^	1.2^***^	0.978^***^		0.23^***^	0.333
ADA^2^			−0.142^**^			−0.099^**^	−0.09^***^			−0.013
*Mediator*										
Self-efficacy							0.668^***^			
*R* ^2^	0.03	0.072	0.125	0.045	0.325	0.368	0.603	0.085	0.222	0.223
*R*^2^ change	0.03	0.043	0.053	0.045	0.280	0.043	0.236	0.085	0.137	0.001
*F*-value	0.778	1.689	2.691^**^	1.278	11.13^***^	11.701^***^	27.056^***^	2.513^*^	6.602^***^	5.776^***^

^*^p < 0.05; ^**^p < 0.01; ^***^p < 0.001; “ADA” is algorithmic decision autonomy.

##### Discussion

Study 1 shows the supporting evidence of the inverted U-shaped hypothesis of H1a, H1b, H1c, and H1, that is, different roles of algorithm agents have significantly different effects on consumers’ purchase decisions. Compared with higher and lower algorithmic decision-making autonomy, when the algorithmic decision-making is a collaborative assistant (the autonomy is at a middle level), the impact of consumers’ purchase decisions is the highest, followed by the other two, and the two slopes are positive first and then negative, thus showing an inverted U-shaped relationship between the impact of algorithmic decision autonomy on the consumer purchase decision.

### Study 2: Mediating effect of self-efficacy

#### Pre-study 2

##### Stimuli and design

We conducted pre-study 2 against the background of the decision to purchase a home AI service robot. Using a one-way between-groups experimental design to describe the materials of three different algorithm decision-making autonomy of home AI robot. A total of 80 participants were recruited through an online experimental platform, and they were randomly divided into three groups (home AI robot algorithm decision-making autonomy is high vs. middle vs. low, and the proportion of the three groups of subjects is 27:27:26). After the manipulation, as in study 1, the effectiveness of the manipulation of the was measured by four algorithmic decision autonomy questions and the overall manipulation judgment question “According to the decision-making scene, do you think its role belongs to a pure performer pure performer/co-assistant/dictatorial substitute?” is asked to give an overall judgment on the role of the algorithm agent by participants (Lucia-Palacios and Pérez-López, 2021). In addition, an attention item was set to test whether the subjects answered carefully. After the experiment, the participants were given a certain reward.

##### Results

ANOVA analysis showed that there was a significant difference between the subjects’ autonomy of algorithmic decision-making for the three groups (*F* (2, 77) = 65.754, *p* < 0.001), and the cross-tabulation analysis of manipulation judgment questions between the experimental groups and the overall algorithmic agent role showed that participants with high autonomy of algorithmic decision-making preferred the algorithm to the role of “dictatorial substitute” role (85.2%); participants with moderate algorithmic decision autonomy preferred the role of “co-assistant” (96.3%); participants with low algorithmic decision autonomy preferred the role of “pure executor” (88.5%). The Pearson chi-square test and Monte Carlo two-tailed test were both significant. *Post hoc* multiple comparison analysis Dunnett’s two-tailed *t*-test showed significant differences between the two groups (*p* = 0 < 0.05), indicating that the experimental manipulation test was successful and that the experimental context and information will be used in study 2.

#### Study 2

##### Study design and procedures

Study 2 used a one-way between-group experimental design (algorithmic decision autonomy high vs. middle vs. low). Two dimensions of consumer purchase decisions, decision quality and decision satisfaction (Amason, 1996; Ameen et al., 2021), and self-efficacy (Köhler et al., 2011), were added. All were measured on a seven-point Likert scale. The procedures and content of the rest of the formal experiment were identical to the Pre-study. As in Study 1, the sample size was measured before the formal experiment, and the calculated sample size should reach more than 159. Therefore, 170 participants were recruited through the Credamo platform for this study, and participants were randomly assigned to 3 groups. The sample distribution of each group was *N*_high_ = 57, *N*_middle_ = 57, *N*_low_ = 56 (female, 65.3%); the proportion of the age group was concentrated between 18 and 50 years old about 90%, and the education level was concentrated in the undergraduate level (71.2%); and the occupational distribution, the majority of the respondents were students, managers, clerical and administrative personnel, etc., and the occupational distribution was relatively even; more than half (69.4%) of the subjects’ monthly income exceeding RMB 3,000, so the valid sample composition is reasonable.

##### Results

First, direct effects were tested. The results of ANOVA analysis with purchase decisions as the dependent variable showed that the effect of algorithmic decision autonomy on purchase decisions was significant (*F* (2, 167) = 21.072, *p* < 0.001). Meanwhile, when algorithmic decision autonomy was at a middle level, the impact of consumer purchase decision evaluation was largest, followed by the other two (*M*_middle_ = 5.511 > *M*_high_ = 5.206 > *M*_low_ = 4.246), the mean equality robustness test was significant, and the Dunnett’s *t* two-tailed test showed significant differences between the two groups (*p* = 0 < 0.05), which confirmed the H1b again.

**Figure 3:**
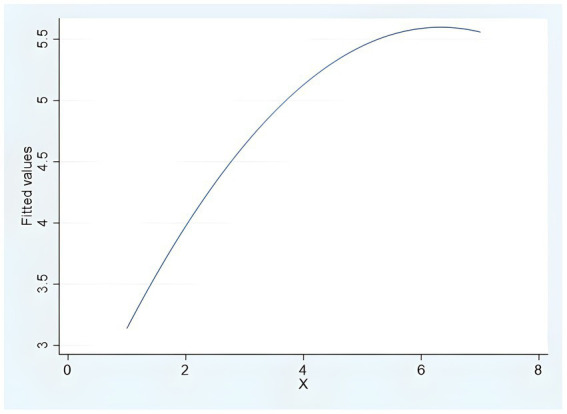
Inverted U-shaped curve fitting (study 2).

Next, the inverted U-shaped effect of algorithmic decision autonomy on consumer purchase decisions is verified. First, the regression estimation fit of the curves was performed, and the results showed that the quadratic and primary terms standardized regression coefficients of *β*_2_ = −0.969 (*t* = −2.891, *p* = 0.004) and *β*_1_ = 1.481 (*t* = 4.419, *p* = 0.000) were significant, while the cubic curve fitting was not significant, proving that the non-“S” curve (the fitted Figure 3), the regression equation for the inverted U-curve relationship can be initially verified: *y* = −0.087(*X*^2^) + 1.096*X* + 2.131. The independent variable was squared and included in the hierarchical regression, and Models 4–7 in Table 1 show that the square of decision autonomy (Model 6, *β*_2_ = −0.099, *p* < 0.01) was significantly negatively correlated with purchase decisions, and the square of algorithmic decision autonomy remained significantly negatively correlated with purchase decision after adding the self-efficacy (Model 7, *β*_2_ = −0.090, *p* < 0.001). After standardization, algorithmic decision autonomy ranges from −1.992 to 1.704, when at −1.992, the slope is 5.341, which is positive; and at 1.704 is −1.822, which is negative. After calculation, the turning point is 0.764, which is within the range of algorithmic decision autonomy. Therefore, H1a, H1b, H1c, and H1 are again supported.

##### Mediating effects

Finally, to test the mediating effect of algorithmic decision autonomy on purchase decisions. The results of the hierarchical regression with self-efficacy as the dependent variable in Table 1 (Model 9–10) show that there is a significant positive effect of algorithmic decision autonomy on self-efficacy (Model 9, *β* = 0.230, *p* < 0.001). None of the coefficients are significant after adding the quadratic of algorithmic decision-making (Model 10), which shows that there is no inverted U-shaped relationship, there is only a linear positive effect, and hypothesis H2 is supported. Meanwhile, self-efficacy has a significant positive effect on consumer purchase decisions (Model 7, *β* = 0.668, *p* < 0.001), and H3 is supported.

To further obtain a more accurate mediating effect of self-efficacy, we refer to the research method recommended by Lin and Feng (2022) on curve effect in management and use the Bootstrap curve mediating test to obtain confidence intervals (Lin and Feng, 2022). Using the MEDCURVE in SPSS with sampling set at 5,000 times and covariates are added, the results show that the total effect of the model is significant, the 95% confidence interval for the bias corrected bootstrap confidence interval for instantaneous indirect effect is [0.0879, 0.2436], which does not contain 0, and the value of the instantaneous indirect effect is 0.1535, indicating that the mediating effect is significant. In addition, the results of the Bootstrap mediating effect test show that (Table 2) the total effect of the model is significant, the effect value is 0.398, and the 95% confidence interval is [0.3019, 0.4937], excluding 0; The direct effect of algorithmic decision autonomy on consumer purchase decisions is significant, the effect value is 0.2421, and the 95% confidence interval is [0.1587, 0.3256], excluding 0; the mediating effect value of algorithmic decision autonomy on purchase decision through self-efficacy is 0.1557, Bootstrap 95% confidence interval is [0.0793, 0.2427], does not contain 0, and the mediating effect percentage is 39.140%, which is an incomplete mediation. In summary, it is indicated that self-efficacy plays a partially mediating role in the process of the influence of algorithmic decision autonomy on purchase decisions, which corroborates the H4.

**Table 2:** Mediating effects of self-efficacy (*N* = 170).

Effects type	Specific paths	Effect value	Standard error	95% confidence intervals	
				Lower CI	Upper CI
Total effect	/	0.3978	0.0486	0.3019	0.4937
Direct effect	ADA-PD^a^	0.2421	0.0423	0.1587	0.3256
Indirect effect	ADA-SE-PD^b^	0.1557	0.0415	0.0793	0.2427

a Algorithmic decision autonomy—purchase decisions.

b Algorithmic decision autonomy—self-efficacy—purchase decisions.

##### Discussion

Study 2 shows that algorithmic decision autonomy has a positive effect on consumers’ self-efficacy, self-efficacy has a positive effect on consumers’ purchase decisions, and self-efficacy partially mediates the inverted-U relationship between algorithmic decision autonomy and purchase decisions. In fact, this can also be reflected in the results of the hierarchical regression, because Models 6–7 in Table 1 show that the square of algorithmic decision autonomy is significantly negatively correlated with purchase decisions, but the correlation coefficient decreases. However, consumers with different power distance perceptions have inherently different preferences for decision-making, which is a prerequisite to influencing self-efficacy. Therefore, in the next study, we will explore the moderating effect of consumer power distance.

### Study 3: Moderating effect of power distance

#### Pre-study 3

##### Study design and procedures

This experiment used a 3 (algorithmic decision autonomy: high vs. middle vs. low) × 2 (power distance: high vs. low) between-group experimental design. 130 participants were recruited for the pre-experiment, and the participants’ sense of power distance was first manipulated experimentally. Using role imagination to realize the manipulation of power distance (Rucker et al., 2014). According to the process control design of the data collection platform, participants are randomly entered into a scenario where participants are told to imagine themselves as the manager or employee of a company while reading a description of a role (Rucker et al., 2012), corresponding to high and low power distances, respectively. Referring to the eight measures proposed by Anderson for the manipulation test (including four reverse measures; Anderson et al., 2012). Then participants were randomly assigned to three different algorithmic decision autonomy scenarios by the platform. The testing procedure of the experiments is similar to studies 1 and 2, but to improve the generalizability and external validity of this study, the stimulus materials are based on an AI shopping guide service program of an e-commerce platform.

##### Results

ANOVA analysis results show that there is a significant difference in the subjects’ decision-making autonomy of the three groups of algorithms (*F* (2, 127) = 174.624, *p* < 0.001). The cross-table analysis between the experimental group of algorithm decision-making autonomy and the overall algorithm agent role shows that participants with high autonomy in algorithmic decision-making preferred the role of “dictatorial substitute” (72.1%); participants with middle autonomy preferred the role of “co-assistant” (97.7%); participants with low autonomy preferred the role of “pure executor” (84.1%). The Pearson chi-square test and Monte Carlo two-tailed test are significant, and the post hoc multiple comparison analysis Dunnett’s *t*-test (two tailed) test showed that there was a significant difference between the two groups (*p* = 0 < 0.05). The results of power distance manipulation showed that the high-power distance group was significantly higher than the low power distance group (*M*_high_ = 6.018, *M*_low_ = 2.528, *F* (1, 128) = 397.643, *p* = 0). It shows that the experiments were successful in manipulating algorithmic decision autonomy and power distance, and the experimental context and information will be used in the formal experiment.

#### Study 3

##### Stimuli and design

The purpose of Study 3 was to examine whether the effect of algorithmic decision autonomy on consumer self-efficacy varies depending on individual power distance and to explore the moderating effect of consumer power distance. The formal experiment was fully consistent with the pretest, and the scales were consistent with Study 1 and 2 (see the Supplementary material), Cronbach’s *α* was above 0.9. A total of 180 participants participated in the experiment, and participants were randomly assigned to six groups. The sample distribution of each group was *N*_high ADA_ = 59, *N*_middle ADA_ = 61, *N*_low ADA_ = 60; *N*_high PD_ = 89, *N*_low PD_ = 91 (ADA: algorithmic decision autonomy; PD: power distance). The proportion of females is 63.3%; the age group is concentrated in the 18–30 years old, accounting for about 92.8%; and the education level is concentrated in the undergraduate stage, accounting for 78.3%; The occupation distribution is relatively uniform, and the basic information composition of effective samples is reasonable.

##### Results

**Figure 4:**
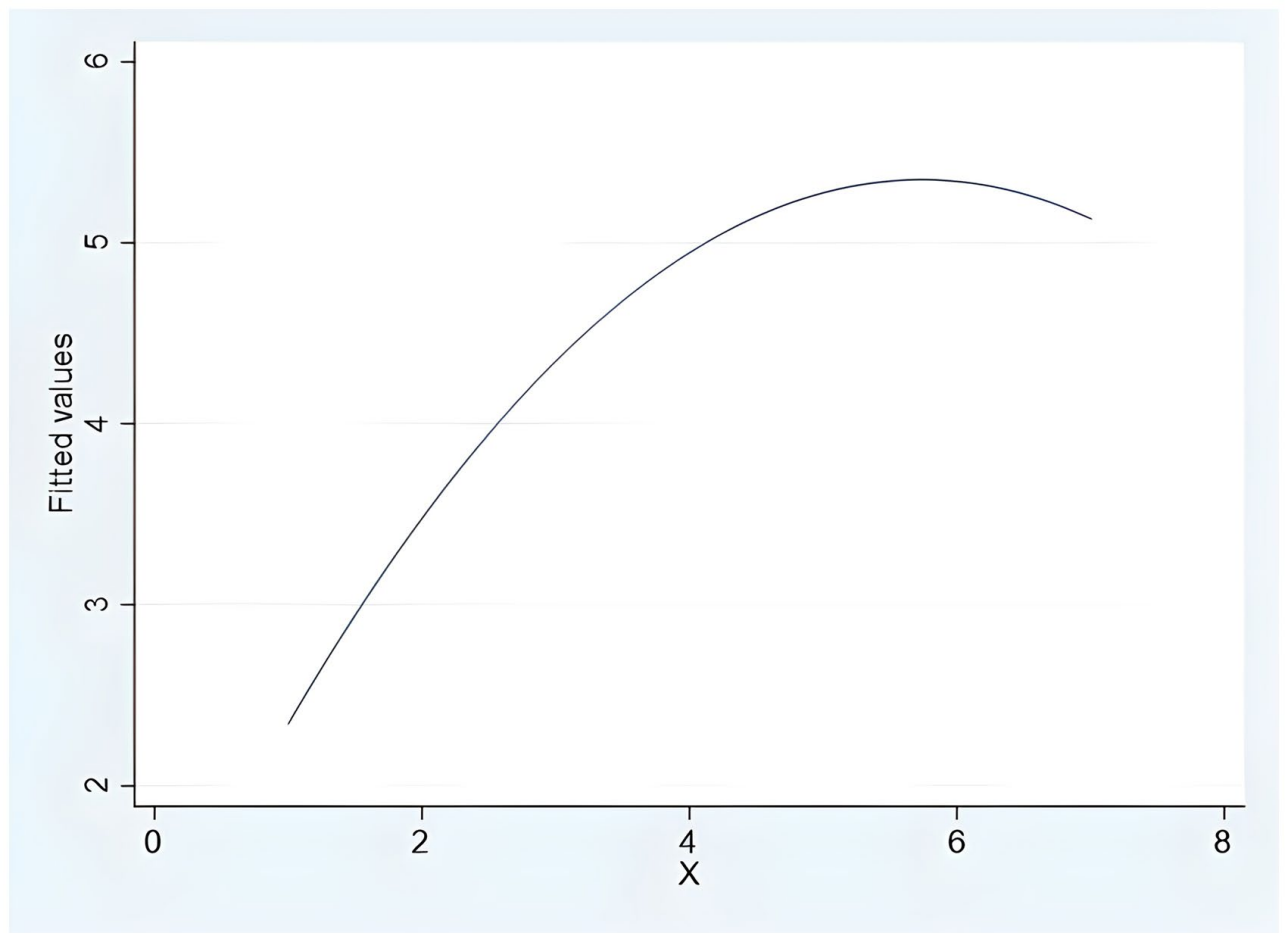
Inverted U-shaped curve fitting (Study 3).

First, is the direct effect test. The results of ANOVA analysis with purchase decisions as the dependent variable show that the effect of algorithmic decision autonomy on purchase decisions is significant (*F* (2, 177) =104.252, *p* < 0.001), while the impact of consumer purchase decision evaluation is largest when algorithmic decision autonomy is at a middle level, followed by the other two (*M*_middle_ = 5.592 > *M*_high_ = 5.057 > *M*_low_ = 2.983). The mean value equality robust test is significant, and the variance homogeneity is significant. The Dunnett’s two tailed *t*-test of post hoc multiple comparative analysis shows that there are significant differences between the two groups (*p* < 0.05). The fitting results of curve regression estimation show that the standardized regression coefficients of quadratic and primary terms are *β*_2_ = −1.322(*t* = −3.640, *p* = 0.000), *β*_1_ = 1.851 (*t* = 5.097, *p* = 0.000) are significant (the fitted Figure 4), and the inverted U-shaped curve regression equation can be obtained: *y* = −0.134 (*x*^2^) + 1.538*x* + 0.941. The independent variables are squared and included in the hierarchical regression, and Model 3 in Table 2 shows that the quadratic and primary terms of the algorithm decision-making autonomy are significantly correlated with the purchase decisions (Model3, *β*_2_ = −0.151, *β*_1_ = 1.668, *p* < 0.001), and H1 is verified.

##### Moderating effects

As can be seen from Table 3, when the moderating and mediating variables and their interaction terms are added in turn, the coefficients of the quadratic terms are not significant (model 4–8), so the moderating variables do not have a significant moderating effect on the inverted U-shaped relationship. The results of the hierarchical regression with self-efficacy as the dependent variable (models 9–14) show, that the coefficients of the quadratic terms and their interaction terms are also not significant when the moderating variables and their interaction terms were added in turn. So, the moderating variables do not have a significant moderating effect on the inverted U-shaped relationship. However, a linear moderating relationship can be found in models 10–13, because the primary term coefficient and interaction are significant (model 13), so there is a linear moderating effect.

**Table 3:** Regression results.

Variables	Purchase decisions (Study 3)								Self-efficacy (Study 3)					
	Model 1	Model 2	Model 3	Model 4	Model 5	Model 6	Model 7	Model 8	Model 9	Model 10	Model 11	Model 12	Model 13	Model 14
*Control variables*														
Gender	0.001	0.048	0.096	0.086	0.086	0.08	0.069	0.084	−0.132	−0.12	−0.065	−0.03	0.022	0.019
Age	−0.032	−0.156	−0.145	−0.157	−0.157	−0.172	−0.097	−0.085	−0.246	−0.277	−0.264	−0.219	−0.122	−0.131
Education	0	0.022	−0.028	−0.028	−0.028	−0.006	−0.002	0.021	0.02	0.026	−0.032	−0.033	−0.02	−0.007
Occupation	0.022	0.006	0.017	0.018	0.018	0.017	−0.02	−0.009	0.048	0.044	0.057	0.054	0.065^*^	0.064^*^
Income	−0.142	−0.02	0.047	0.043	0.043	0.043	0.03	0.011	−0.051	−0.021	0.056	0.071	0.024	0.023
Familiarity	0.275^*^	0.167	0.221^*^	0.218^*^	0.218^*^	0.225^*^	0.012	0.027	0.339^*^	0.312^*^	0.374^**^	0.384^**^	0.365^**^	0.369^**^
*Independent variables*														
ADA		0.452^***^	1.668^***^	1.633^***^	1.632^***^	2.142^**^	0.777	0.25		0.113	1.501^***^	1.634^***^	2.057^***^	2.368^**^
ADA^2^			−0.151^***^	−0.147^***^	−0.147^***^	−0.209^*^	−0.078	−0.023			−0.172^***^	−0.187^***^	−0.19^***^	−0.228^**^
*Moderator*														
PD				0.039	0.038	0.23	0.01	0.12				−0.146^*^	0.265^*^	0.382
ADA × PD					0	−0.124	−0.023	0.096					−0.1^**^	−0.176
ADA^2^ × PD						0.015	0.01	−0.003						0.009
*Mediator*														
SE							0.577^***^	0.889^***^						
SE × PD								−0.069^**^						
*R* ^2^	0.031	0.314	0.371	0.373	0.373	0.375	0.664	0.681	0.052	0.068	0.135	0.165	0.218	0.219
*R*^2^ change	0.031	0.283	0.056	0.002	0	0.002	0.288	0.018	0.052	0.016	0.066	0.03	0.053	0.001
*F*-value	0.927	11.266^***^	12.591^***^	11.239^***^	10.056^***^	9.181^***^	27.49^***^	27.312^***^	1.597	1.803^***^	3.322^***^	3.727^***^	4.717^***^	4.283^***^

^*^p < 0.05; ^**^p < 0.01; ^***^p < 0.001; ADA is algorithmic decision autonomy; PD is power distance; SE is self-efficacy.

##### Moderated mediation analysis

Through the PROCESS provided by Hayes, Model 8 of Bootstrap was selected to further do the mediated model test with moderation, setting the sample size at 5,000 and the confidence interval at 95%, and the regression model results were obtained as shown in Table 4, where it can be found that the moderating effect (interaction term) was significantly negative when self-efficacy was the dependent variable (*β* = −0.098, *p* < 0.01); while the moderating effect (interaction term) was significantly negative (*β* = −0.060, *p* < 0.01) when purchase decisions were the outcome variable. Further results show (Table 5) that the mediating index with moderation was-0.0582, and the bootstrap 95% CI interval is [−0.0987, −0.0171], which did not contain 0, so the mediating effect with moderation was significant. When algorithmic decision autonomy was low, the mediation effect of self-efficacy was 0.1732, the bootstrap 95% CI interval did not contain 0, and the mediation was significant; when algorithmic decision autonomy was high, the mediation effect of self-efficacy is −0.0555, bootstrap 95% CI interval contained 0, and the mediation was not significant; while the contrasts between the effects were all significant.

**Table 4:** Bootstrap regression results.

Variables	Self-efficacy			Purchase decisions		
	Coeff	SE	*t*	Coeff	SE	*t*
*Control variables*						
Gender	−0.046	0.245	−0.187	0.061	0.146	0.417
Age	−0.147	0.171	−0.861	−0.089	0.103	−0.869
Education	0.043	0.262	0.163	−0.005	0.157	−0.032
Occupation	0.051	0.032	1.623	−0.023	0.019	−1.223
Income	−0.062	0.124	−0.499	0.014	0.074	0.192
Familiarity	0.2965^*^	0.131	2.260	−0.011	0.080	−0.140
*Independent variables*						
ADA	0.099	0.064	1.535	0.389^***^	0.039	10.038
*Moderator*						
PD	−0.118^*^	0.059	−1.980	−0.132^***^	0.036	3.678
Int_1	−0.098^**^	0.031	−3.176	−0.060^**^	0.019	3.173
*Mediator*						
M_SE				0.596^***^	0.046	12.984
*R*	0.373			0.812		
*R* ^2^	0.139			0.660		
*F*	3.049^**^			32.786^***^		

^*^p < 0.05; ^**^p < 0.01; ^***^p < 0.001; ADA is algorithmic decision autonomy; PD is power distance; M_SE is self-efficacy; “Int_1” is ADA × PD.

**Table 5:** Moderated mediating effects.

Paths	Effects indicators	Effect INDEX	Boot SE	Boot LLCI	Boot ULCI
Indirect effect (X-M-Y)	Power distance	−0.0582	0.0206	−0.0983	−0.0171	−1.9645 (*M* − SD)	0.1732	0.0532	0.067	0.2729
	(*M* = 0)	0.0588	0.0402	−0.0213	0.1362
	1.9645(*M* + SD)	−0.0555	0.0605	−0.1766	0.0598
Pairwise contrast	eff2–eff1	−0.1144	0.0405	−0.1931	−0.0336
	eff3–eff1	−0.2287	0.0809	−0.3861	−0.0673
	eff3–eff2	−0.1144	0.0405	−0.1931	−0.0336

**Figure 5:**
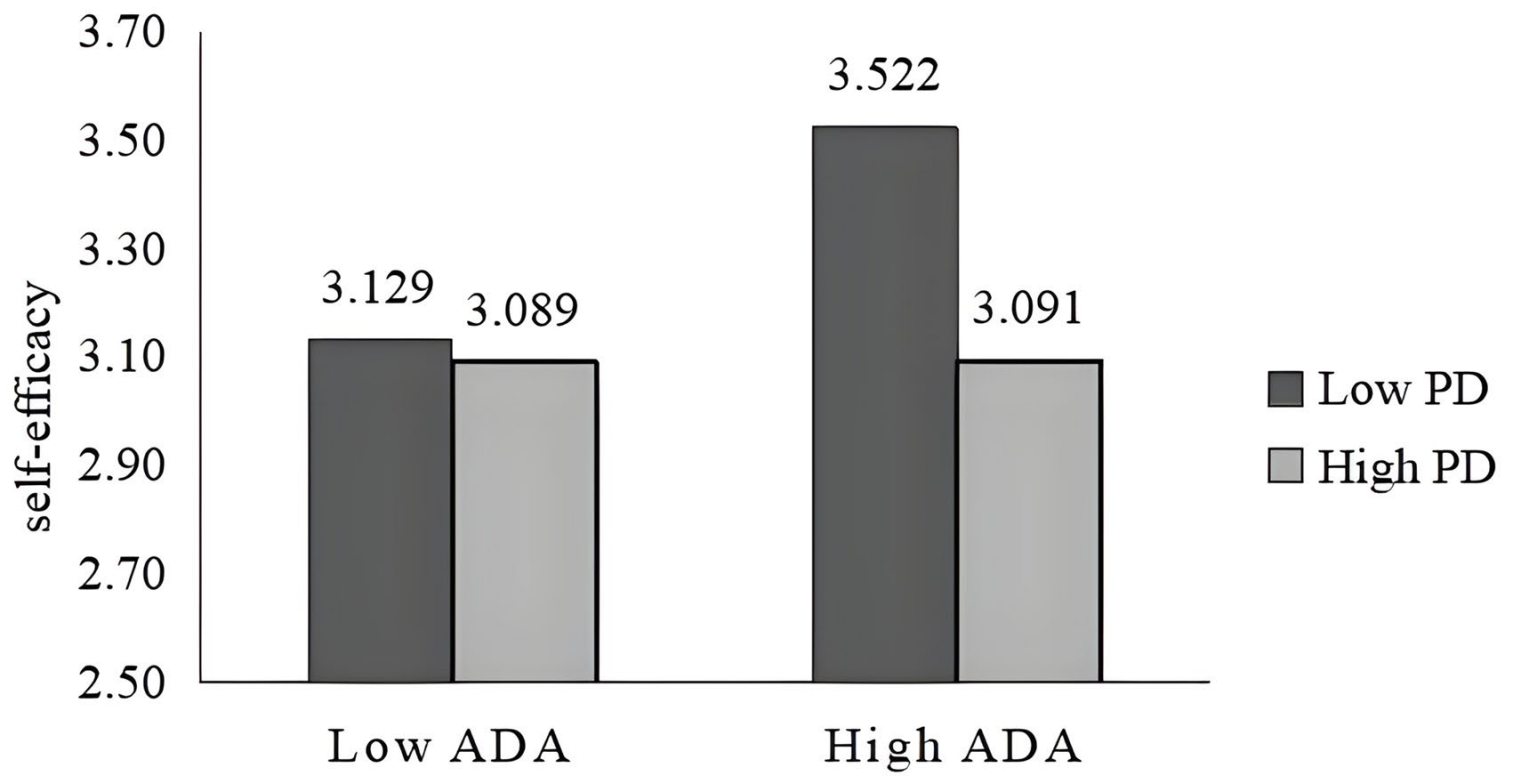
The moderating effect of power distance on self-efficacy.

**Figure 6:**
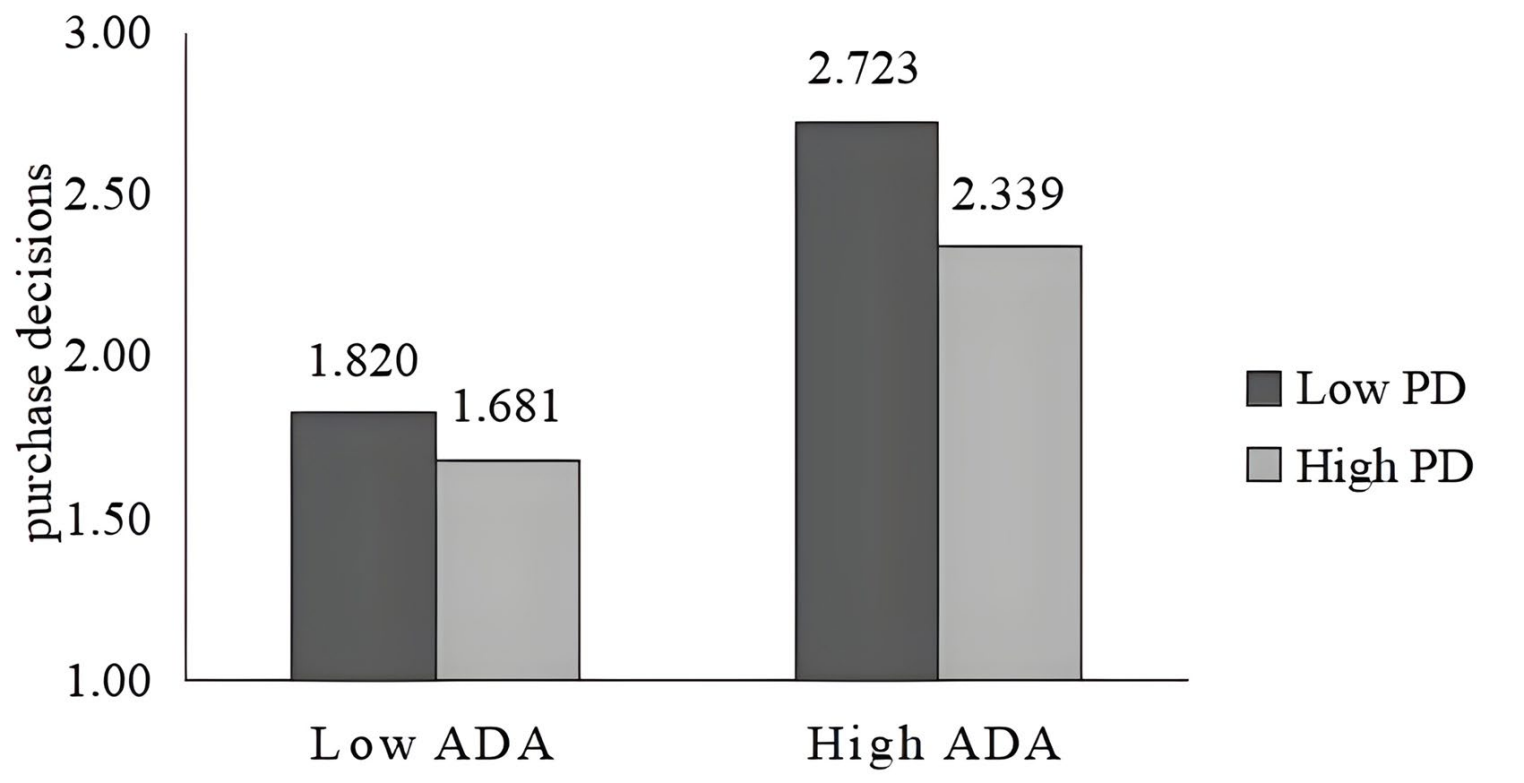
The oderating effect of power distance on purchasing decisions.

To visualize the moderating effect of power distance, the moderating effects of power distance on self-efficacy and purchase decisions are plotted in Figures 5, 6. Consumers with low power distance perceive that algorithmic decision autonomy can bring them ease of decision-making, and do not experience algorithmic decision aversion even when the level of algorithmic decision autonomy is high. Whereas, people with high power distance have more status (Kim and Zhang, 2014) and self-confidence and focus on their emotional self-evaluation of being accepted by society so that when the level of algorithmic decision autonomy is high compared to those with a low power distance sense will instead cause them to lose their level of self-determination evaluation, reduce their self-efficacy, and negatively affect the purchase decisions. Also, it can be seen from the figure that algorithmic decision-making varies more with different degrees of autonomy for consumers with low power distance perception. Consumers with low power distance had higher self-efficacy and purchase decision influence than those with high power distance at the same level of autonomy.

##### Discussion

The results of Study 3 support H5 and H6 that power distance moderates the effect of algorithmic decision autonomy on consumers’ self-efficacy and purchase decisions, but not on the inverted U-shaped relationship. Because people with high power distance have higher self-efficacy: autonomy, competence, and relationship. When algorithmic decision autonomy is high, artificial intelligence substitutes for making decisions, and the role of algorithmic agents as “dictatorial substitutes” will instead threaten the self-efficacy of the high-power distance, cannot reflect their superiority, and the evaluation of the purchase decisions will be correspondingly low.

## General discussion

The ability to predict consumer behavior and decisions is a must for business success (Struhl, 2017), therefore, more and more companies are using algorithms to make business decisions that directly affect potential and existing customers (Yalcin et al., 2022), using algorithms to collect and process information about data generated by consumers during shopping activities to make automated decisions about data analytics (Helbing et al., 2018), driving a shift from descriptive to predictive models for algorithmic data analysis. However, various problems arising from the use of autonomous algorithm decision-making also make people face the risks and challenges it brings, and even cause humans to lose control of it (Günther et al., 2017). Peter F. Drucker also warned that “unless we control the new power extended by knowledge, it is difficult for humans to continue to survive.” Therefore, our research provides support for understanding consumers’ responses to algorithmic decision-making and also provides insights and management recommendations to address this phenomenon.

### Conclusion

Through three studies, we verify the consumer’s response to different algorithmic decision autonomy. Study 1 confirmed the inverted U-shaped effect of algorithmic decision autonomy on consumer purchase decisions, that is, lower levels of algorithmic decision autonomy have a negative influence on consumer purchase decisions; when algorithmic decision autonomy is at a middle level, which is the most comfortable agent relationship, it reaches the largest influence on consumer purchase decisions; and when algorithmic decision autonomy is high, it will have a negative influence on consumer purchase decisions. Study 2 explored the mediating mechanism of algorithmic decision autonomy on consumers’ purchase decisions, that is, algorithmic decision autonomy has a positive effect on consumer self-efficacy, which in turn has a positive effect on consumer purchase decisions, ultimately, self-efficacy partially mediates the inverted U relationship between algorithmic decision autonomy and purchase decisions. Study 3 showed that the effect of algorithmic decision autonomy on consumer purchase decisions varies depending on the consumer’s sense of power distance. For consumers with a low power distance, the effect of algorithmic decision autonomy on consumer purchase decisions was more significant than for consumers with a high-power distance.

### Theoretical implications

The theoretical implications of this research are: Firstly, we provide a clear and detailed definition and classification of AI algorithmic decision autonomy and conduct the first empirical study to investigate the inverted U-shaped impact of algorithmic decision autonomy on consumer purchase decisions. The findings further illustrate the differences in the impact of different degrees of algorithmic decision autonomy on consumer purchase decisions. Previous studies have argued for either algorithmic appreciation (Banker and Khetani, 2019; Logg et al., 2019) or algorithmic aversion (Dietvorst et al., 2015; Zhang et al., 2022) of algorithmic decision-making, but the impact of algorithmic decision autonomy on consumer purchase decisions has not been fully explored. We investigated the inverted U-shaped influence of algorithmic decision autonomy on consumer purchase decisions. In other words, there is an optimal state of influence of algorithmic decision autonomy on consumer purchase decisions: as the degree of algorithmic decision autonomy gradually increases from low to high, the quality of consumer purchase decisions and decision satisfaction show a trend of first increasing and then decreasing. Algorithmic decision-making belongs to the role of middle-level collaborative decision-maker, which is better than low-level pure executors and high-level autonomous dictatorial substitute.

Secondly, the research enriches the potential mechanism of the relationship between algorithmic decision autonomy and consumer purchase decisions and contributes to the exploration of the impact mechanism of algorithmic decisions on consumer behavior. Previous studies usually take psychological resistance, identity threat, privacy concerns, trust, or consumer’s empathy for algorithms as mediating mechanisms. Based on the self-determination theory, our research, from the perspective of consumers, has clearly defined consumers’ autonomy in algorithm decision-making and explored the mediating mechanism of self-efficacy based on previous work on consumers’ different perceptions of humans and algorithms.

Thirdly, we investigate an important factor that influences consumer responses to the autonomy of different algorithmic decisions: the power distance. Previous studies have mostly explored the moderating role of algorithmic anthropomorphic features, communication style, or subjective and objective task characteristics when deploying algorithms from the perspective of AI itself. In this paper, we explain the boundary effects of algorithmic decision autonomy on purchase decisions by introducing power distance perception as a moderating variable and providing insight into other possible moderation.

Finally, this research also enriches the literature related to AI algorithmic marketing. Although algorithmic decision-making is prevalent in the marketing environment, current research on users’ psychological and behavioral responses in AI marketing is mostly qualitative, such as interview research and conceptual model construction, and lacks analytical support of empirical data and in-depth exploration of mechanisms. However, understanding the psychological mechanisms and behavioral attitudes of consumers toward AI services and purchase decisions, as well as how to design AI for consumers to quickly accept this shift in service format, are issues that should be of urgent concern to scholars in the current marketing field.

### Practical implications

The findings of this research have rich managerial implications. First, our research shows the inverted U-shaped effect of algorithmic decision autonomy on consumer purchase decisions. Therefore, for companies, there should be a “degree” of autonomy in using algorithmic decision-making, and “overdoing it” should be avoided. While adapting to the digital management trend, companies should adopt the appropriate role of algorithmic agents to serve the consumer’s decision, and not let the algorithmic decision make the human perceive the loss of autonomy. The philosopher Kant said that “human autonomy leads to free behavior, and the absence of autonomy means the partial disintegration of freedom and the complete disintegration of morality.” Therefore, companies should explore personalized algorithm design to highlight human control over algorithmic decision-making, and giving consumers a sense of self-control and autonomy in the purchase decision process may be a better choice.

Second, research shows that consumer self-efficacy plays a mediating role between algorithmic decision-making autonomy and purchase decisions, so companies should focus on enhancing users’ self-efficacy. For example, by enhancing the sense of participation and co-creation in the user’s algorithmic decision-making process, consumers can perceive their existence as unique individuals who have not lost their absolute dominant decision-making power in the algorithmic decision-making process. During the purchase decision task, set up techniques and corresponding guidance procedures to stimulate customers’ self-efficacy and make them believe that they can cope with various challenges in the purchase decision process.

Third, due to the negative moderating effect of power distance, retailers should form a portrait of different power distance consumers by using big data analysis to adopt different degrees of autonomy in algorithmic decision-making. For consumers with high power distance, a lower degree of algorithmic decision autonomy should be adopted because people with high power distance follow the dynamic orientation of power in making decisions for themselves, value their value and importance, and focus more on themselves, so a low degree of algorithmic autonomy will increase consumers’ sense of self-judgment and self-determination and satisfy their sense of self-efficacy; while for consumers with low power distance, they should be guided through oriented steps for proper guidance, enough to stimulate customers’ self-efficacy and improve decision evaluation.

Finally, retail enterprises can establish an algorithm application impact assessment system and build a perfect user feedback mechanism. For typical scenarios, enterprises should assess the impact of algorithms on consumers’ interests and individuals’ basic rights before the algorithms are formally launched, take corresponding preventive measures for the relevant risks found in the assessment; and establish an algorithm transparency system to disclose information related to the adoption of algorithms to relevant departments and the public, including the purpose of adoption, application scenarios, and technical implementation of algorithms, etc. For those algorithm decisions that may have a significant impact on individuals and society, companies should explain the basic principles of the algorithms. Giving individuals the right to redress for algorithmic damages through algorithmic application rules, and allowing individuals to dispute algorithmic decisions and conduct manual reviews, etc. (Burton et al., 2020).

### Limitations and future research

Firstly, this research only considers the division of algorithmic autonomy and does not distinguish between algorithmic decision task types, whereas research has shown that people are more reluctant to use algorithms in more subjective tasks, and decision tasks are governed by personal tastes (Yeomans et al., 2019), so future research could consider the triadic interaction of task type, algorithmic decision autonomy, and consumers’ individual characteristics to explore the behavioral outcomes of consumer decisions under the role of more contexts.

Secondly, this research does not conduct follow-up research on the results of consumer algorithm decision-making behavior, research has shown that algorithms can have a significant impact on consumers’ brand attitudes, and thus future research could explore the chain-mediating mechanisms of consumers’ self-efficacy and purchase decision evaluation on consumers’ brand attitudes in terms of algorithmic decision autonomy (Srinivasan and Sarial-Abi, 2021).

Finally, research by Martin and Waldman suggests that as decision importance increases, individuals’ perceptions of the legitimacy of using an algorithm to make a decision decrease (Martin and Waldman, 2022). Therefore, future research could examine the role of decision importance and how to improve consumers’ decision experiences.

## Data availability statement

The original contributions presented in the study are included in the article/Supplementary material, further inquiries can be directed to the corresponding author.

## Author contributions

FY supervised the study and performed a thorough review and revision of the manuscript. LX designed the study, analyzed the data, and wrote the manuscript. All authors contributed equally to this manuscript, reviewed, and approved this manuscript for publication.

## Funding

This work was supported by grants from the National Social Science Foundation of China (no: 18BJY167).

## Conflict of interest

The authors declare that the research was conducted in the absence of any commercial or financial relationships that could be construed as a potential conflict of interest.

## Publisher’s note

All claims expressed in this article are solely those of the authors and do not necessarily represent those of their affiliated organizations, or those of the publisher, the editors and the reviewers. Any product that may be evaluated in this article, or claim that may be made by its manufacturer, is not guaranteed or endorsed by the publisher.

## Footnotes

^1^The rise of algorithmic decision making: Some ethical issues and strategies in the age of artificial intelligence|AI watch (2017). www.sohu.com. Available at: https://www.sohu.com/a/143165651_455313 (Accessed June 19, 2022).
