# Supplementary material for: Exploring the role of AI algorithmic agents: The impact of algorithmic decision autonomy on consumer purchase decisions
Source: Front Psychol. 2022 Oct 20;13:1009173. doi: 10.3389/fpsyg.2022.1009173 (PMC9630471; doi:10.3389/fpsyg.2022.1009173)
Supplement: Supplementary file 1 [file Data_Sheet_1.docx]

Supplementary Material

**Experimental materials**

**Study1：(Algorithm Recommendation News)**

A news information distribution platform that makes the following news information decisions.

**High autonomy of algorithm decision (Group A scenario):** The AI algorithm system of this news and information platform summarizes preferences based on your historical click browsing records, recommends the most similar news to you, and uses groups with similar interests to make similar news recommendations, as well as recommending the most popular news with the most clicks to users, dominating the amount, type and preference of the news audience to get news, and you can only accept the system to push decisions without any choice manipulation.

**Middle autonomy of algorithm decision (Group B scenario):** The news information platform's AI algorithm system summarizes preferences based on your historical click browsing records, recommends the news with the highest similarity to you, and uses groups with similar interests to make similar news recommendations, as well as recommending the most popular news with the most clicks to users, and supports self-setting news filtering filter function push to assist you in making news selection browsing decisions.

**Low autonomy of algorithm decision (Group C scenario):** The AI news information platform only does some mechanical news reporting work every day according to the established procedures, and will not actively push relevant news to you, only based on your search at each step to display, you need to make your own relevant news search decisions.

**Study2: (Suppose you want to buy a home service robot)**

**High autonomy of algorithm decision (Group A scenario):** “Huami” is a home service robot that excels in speed, data storage, and processing power, moves around the home, opens and closes doors, searches for information, sets calendars and reminders, and connects and controls other home electronics, automatically recognizes and records data about the user's habits in daily life, and automatically enables functions and makes decisions, even on important functions, without your consent.

**Middle autonomy of algorithm decision (Group B scenario):** “Huami” is a home service robot that offers superior performance in terms of speed, data storage, and processing power, moves around the home, opens and closes doors, searches for information, sets calendars and reminders, and connects to and controls other home electronics, automatically recognizes and records data about the user's habits in daily life, activates relevant functions for decision making with your authorized consent, and provides you with reference suggestions to assist you in your decision making

**Low autonomy of algorithm decision (Group C scenario):** The “Huami” home service robot has an excellent performance in terms of speed, data storage, and processing power, and can move around the home space to open and close doors, search for information, set calendars and reminders, and connect and control other home electronic devices, etc. It can automatically recognize and record and analyze data about the user's daily habits, but the start-up function will only do some standardized and repetitive work according to the established procedures, and will not provide you with reference suggestions.

**Study3：AI shopping guide service program**

**High autonomy of algorithm decision (Group A scenario):** An e-commerce platform has launched an AI intelligent shopping guide service program, which combines your browsing records, purchase transaction records and other data through algorithms, and even supports tone analysis functions, allowing AI guides to recognize emotions and so on, replacing you with independent search, dominating the type and preference of goods you acquire, and making shopping decisions instead of you.

**Middle autonomy of algorithm decision (Group B scenario):** An e-commerce platform has launched an AI intelligent shopping guide service program that combines your browsing history, purchase transaction records and in-store receipts, preferences and other data through an algorithm to provide you with personalized quotes, portfolio opinions and other analysis for your reference, assisting you in your product search and helping you make decisions.

**Low autonomy of algorithm decision (Group C scenario):** An e-commerce platform launched an AI intelligent shopping guide service program, but the AI guide will only follow the established algorithm program to do some standardized repetitive work, the degree of intelligence is low, not very flexible, not able to adapt to respond to customer needs, based entirely on the need for you to search.

**Autonomy manipulation test:**

The extent to which ......

it can decide for itself how to perform its tasks;

it can make its own decisions;

It can take initiatives on its own;

it can work independently on its own;

Based on the above purchase decision scenario, do you think its role is: **a pure performer / co-assistant / dictatorial substitute** to you

**Purchase decisions**

Decision quality：

1. The decision quality is high；
2. The quality of decisions is better than I expected;
3. Decision making very favorable to my goals;
4. I have great confidence in its decision-making.

Decision satisfaction：

1. I would love to see such a decision being implemented;
2. I am most satisfied with such a decision;
3. I am very willing to accept such a decision;
4. I think such a decision is safe and reliable.

**Self-efficacy**

1. I believe that the application of the algorithm can make me better complete the decision;
2. I believe I can master the ability to use algorithms to meet decision needs;
3. I believe I can use the algorithm to reach my decision goals as much as I want;
4. I’m sure I can make good use of the algorithm to meet my decision needs.

**Manipulation of power distance：**

The research used a role imagery approach to achieve manipulation of power distance. Participants were told to imagine themselves as the owner or employee of a company when reading a description of a role

**Low power distance**: As an **employee**, the boss of the company uses the algorithm to assign you tasks, you are responsible for carrying out the orders of the algorithm, and the algorithm can determine the flow of your work and evaluate your work based on relevant work indicators, as an employee you must comply with the company and the algorithm’s decision-making instructions, the algorithm will determine your salary performance remittance and total to the boss based on the monthly evaluation of you, you will not see your boss’s evaluation of you, and you have no opportunity to evaluate your boss.

**High power distance**: As a **boss**, you are responsible for directing the creation of different algorithmic products and managing the work. You can determine the workflow of your employees through the relevant algorithmic procedures and evaluate their work based on the relevant work indicators. Employees must comply with the company's and the algorithm's decision-making instructions, employees never see your evaluations, and employees do not have the opportunity to evaluate you.

Manipulation tests: In this role scenario

1. I can get him/her/them to listen to what I say.

2. My wishes do not carry much weight. (r)

3. I can get him/her/them to do what I want.

4. Even if I voice them, my views have little sway. (r)

5. I think I have a great deal of power.

6. My ideas and opinions are often ignored. (r)

7. Even when I try, I am not able to get my way. (r)

8. If I want to, I get to make the decisions.

All of the above measures are on a seven-point Likert scale: how strongly you agree or disagree with each of the following statements (1 = “strongly disagree,” and 7 = “strongly agree”)
